# *Nfatc1*’s Role in Mammary Epithelial Morphogenesis and Basal Stem/progenitor Cell Self-renewal

**DOI:** 10.1007/s10911-021-09502-6

**Published:** 2021-12-21

**Authors:** Melissa McNeil, Yingying Han, Peng Sun, Kazuhide Watanabe, Jun Jiang, Natasha Chen, Zhengquan Yu, Bin Zhou, Xing Dai

**Affiliations:** 1 Department of Biological Chemistry, School of Medicine, University of California, Irvine, CA 92697 USA; 2 State Key Laboratories for Agrobiotechnology, Department of Nutrition and Health, College of Biological Sciences, China Agricultural University, Beijing, 100193 China; 3 Departments of Genetics, Pediatrics, and Medicine (Cardiology), The Wilf Cardiovascular Research Institute, The Institute for Aging Research, Albert Einstein College of Medicine, Bronx, NY 10461 USA

**Keywords:** Mammary gland, Basal cell, Stem cell, *Nfatc1*, Self-renewal

## Abstract

Mammary gland is an outstanding system to study the regulatory mechanisms governing adult epithelial stem cell activity. Stem cells in the basal layer of the mammary gland fuel the morphogenesis and regeneration of a complex epithelial network during development and upon transplantation. The self-renewal of basal stem/progenitor cells is subjected to regulation by both cell-intrinsic and extrinsic mechanisms. Nfatc1 is a transcription factor that regulates breast tumorigenesis and metastasis, but its role in mammary epithelial development and stem cell function has not been investigated. Here we show that *Nfatc1* is expressed in a small subset of mammary basal epithelial cells and its epithelial-specific deletion results in mild defects in side branching and basal-luminal cell balance. Moreover, *Nfatc1*-deficient basal cells exhibit reduced colony forming ability in vitro and somewhat compromised regenerative potential upon transplantation. Thus, our study provides evidence for a detectable yet non-essential role of *Nfatc1* in mammary epithelial morphogenesis and basal stem/progenitor cell self-renewal.

**Supplementary Information:**

The online version contains supplementary material available at 10.1007/s10911-021-09502-6.

## Introduction

The mammary gland is a dynamic and regenerative organ that undergoes most of its development after birth, with dramatic structural and functional changes occurring during puberty, pregnancy, lactation, and involution. Under the regulation of hormones and local growth factors, stem/progenitor cells in the mammary epithelium self-renew, proliferate, and differentiate to drive the growth, remodeling, and regeneration of the bi-lineage epithelial network composed of an outer basal/myoepithelial layer and an inner luminal layer [1–4]. The basal compartment houses both unipotent (generating only basal progenies) and multi/bi-potent (generating both basal and luminal progenies) stem cells that fuel morphogenesis and regeneration [1, 5–9]. Despite extensive studies identifying a myriad of molecular and signaling pathways that regulate mammary epithelial stem cell activity and differentiation [1, 6], transcriptional mechanisms underlying mammary epithelial morphogenesis and basal stem cell self-renewal remain to be fully understood.

Nfatc1 (also known as Nfat2) belongs to the NFAT family of transcription factors, which can be dephosphorylated and activated in response to intracellular calcium and translocate into the nucleus to control target gene expression [10]. Nfatc1 plays important roles in both embryonic and adult stem cells, such as regulating early lineage specification in embryonic stem cells [11], maintaining quiescent hair follicle stem cells [12], and directing lung stem cell differentiation and regeneration [13]. Nfatc1 is also required for breast cancer cell migration and invasion in vitro as well as tumorigenesis and metastasis in vivo, and its expression is upregulated in breast cancer [14–16]. Nfatc2 protein was reported to accumulate in mammary epithelial cells upon treatment with Wnt5b, an inhibitor of mammary epithelial outgrowth, and was proposed to be a nuclear effector of downstream non-canonical Wnt signaling [17]. Like Nfatc1, Nfatc2 also promotes breast cancer cell invasion and metastasis [16, 18]. However, the in vivo function of *Nfat* genes in normal mammary gland development and basal cell self-renewal has not been investigated.

In this study, we show that *Nfatc1* is expressed in a small subset of mammary basal epithelial cells and its deletion from the mammary epithelium results in mild defects in side branching and basal-luminal cell balance. Moreover, we provide evidence for a detectable but non-essential role for *Nfatc1* in basal cell colony formation in vitro and mammary epithelial outgrowth in vivo.

## Results

### *Nfatc1* is Expressed in a Small Subset of Mammary Basal and Luminal Epithelial Cells

**Fig. 1:**
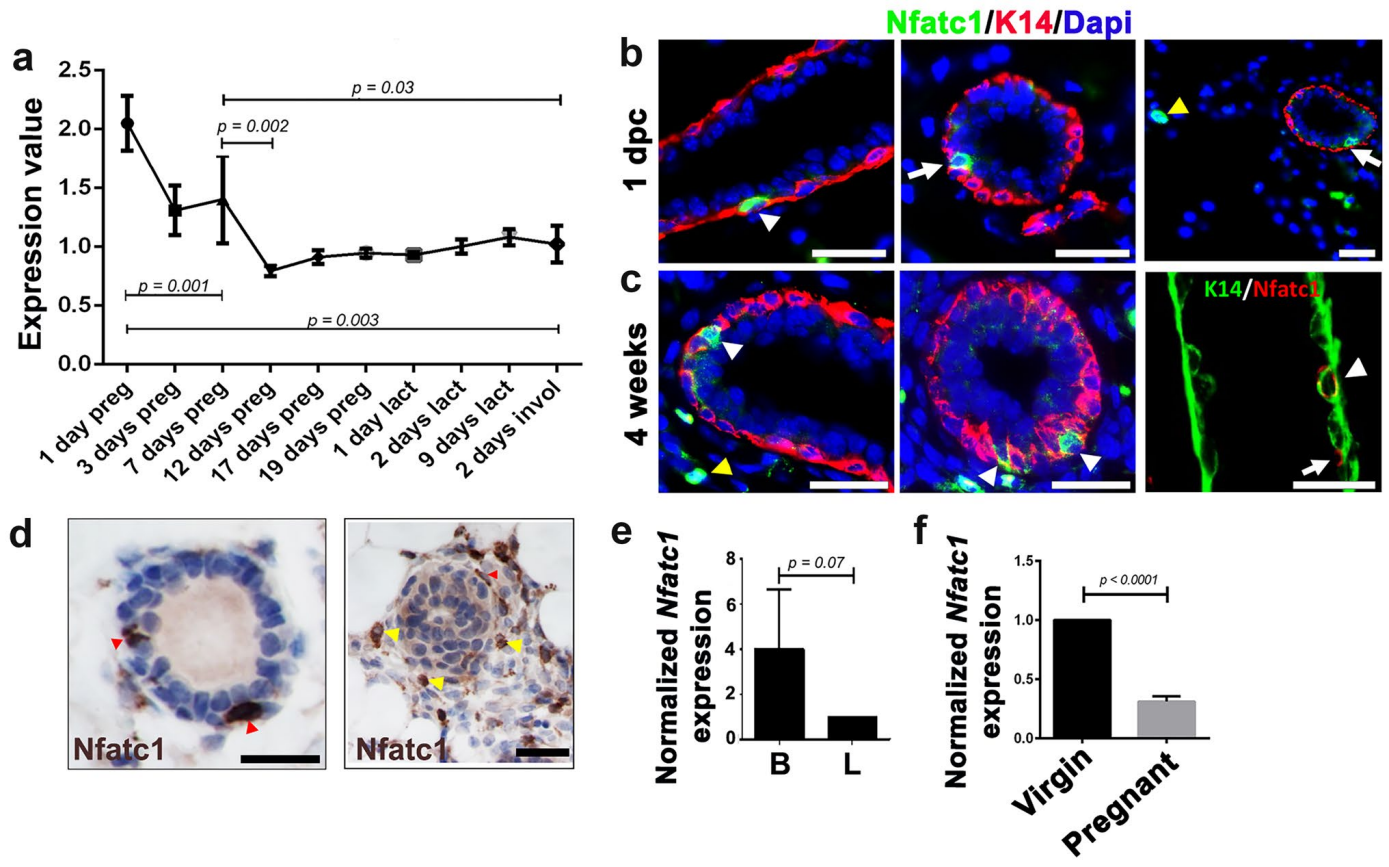
Nfatc1 expression in mouse mammary epithelium. **a**, *Nfatc1* mRNA expression in the mammary gland of wild-type (WT) mice during pregnancy and lactation. Average values of expression were calculated based on raw expression levels of *Nfatc1* mRNA detected using Affymetrix MG_U74Av2 chips per GDS2843/102209_at (NCBI's Gene Expression Omnibus). *1 day preg*, the day a vaginal plug was observed. N = 4 replicate samples for each stage. **b**, Immunofluorescence for Nfatc1 protein in the mammary glands of 1-day-pregnant (1 dpc) WT mice (N = 4). Multiple ducts from different mice are shown. **c-d**, Immunofluorescence (**c**) and immunohistochemistry (**d**) for Nfatc1 protein in the mammary glands of 4-week-old WT mice (N = 3). K14 antibody stains the basal cells, and DAPI stains the nuclei. White/red and yellow arrowheads indicate Nfatc1-positive basal and stromal cells, respectively, whereas arrows indicate Nfatc1-positive luminal cells. Note the presence of Nfatc1-positive cap (red arrowhead) and stromal (yellow arrowheads) cells in the terminal end bud structure (**d**, right). **e**, RT-qPCR on FACS-isolated basal and luminal cells from 8-week-old virgin WT female mice (N = 5 mice analyzed in different sorting experiments). **f**, RT-qPCR on sorted basal cells from 15-week-old virgin and mid-pregnant (P14) WT mice. N = 3 mice for each stage. *GAPDH* gene was used for normalization in **e**–**f**. Scale bar: 50 μm in **b**, **c**, and **d**.

To probe potential *Nfatc1* involvement in mammary epithelial morphogenesis, we first interrogated a publicly available microarray dataset on whole mammary gland tissues [19, 20] (see Materials and Methods). This analysis revealed high *Nfatc1* expression during early pregnancy that gradually declined by mid-pregnancy and remained low during lactation and involution (Fig. 1a). Using immunostaining, we detected Nfatc1 protein in the basal (marked by basal cell marker K14), luminal (inner, K14^−^ layer), and stromal cells of mammary glands from early pregnant mice (Fig. 1b). Nfatc1-positive cells were rare in the basal and luminal compartments, typically at 1–2 cells per cross-section of a single mammary duct (Fig. 1b). Immunofluorescence and immunohistochemistry also detected Nfatc1-positive basal, luminal, and stromal cells in pubertal mammary glands, both in the ducts and in terminal end bud structures (Fig. 1c, d).

We next performed RT-qPCR analysis on FACS-sorted basal and luminal cells of adult virgin mammary glands, which revealed that *Nfatc1* mRNA trended towards being expressed at a higher level in basal cells (Figs. 1e and S1a). This finding is consistent with a previous report detecting elevated *Nfatc1* expression in mammary basal cells [21], as well as with our own RNA-seq data revealing a threefold enrichment of *Nfatc1* mRNA level in basal over luminal cells (*p* < 10^–6^) [22]. Compared to FACS-sorted basal cells from adult virgin glands, *Nfatc1* mRNA expression decreased significantly in basal cells from mammary glands in mid-pregnancy (Figs. 1f and S1a). We also generated *Nfatc1-Cre;ROSA*^*mTmG*^ mice, where *Nfatc1*-expressing cells and their descendants are marked by GFP expression. Flow cytometry-based quantification of GFP fluorescence in conjunction with basal/luminal surface marker immunostaining revealed rare (< 10%) GFP^+^ cells in both the basal and luminal populations of 8-week-old virgin and multiparous 6–9-month-old mice (Fig. S1b, c). Together, our data show dynamic *Nfatc1* expression in a small subset of basal and luminal epithelial cells of the mammary gland across different reproductive stages.

### Epithelium-Specific Deletion of *Nfatc1* Results in Mild Reductions in Alveolar Bud Formation/Side Branching and Basal-to-Luminal Cell Ratio

**Fig. 2:**
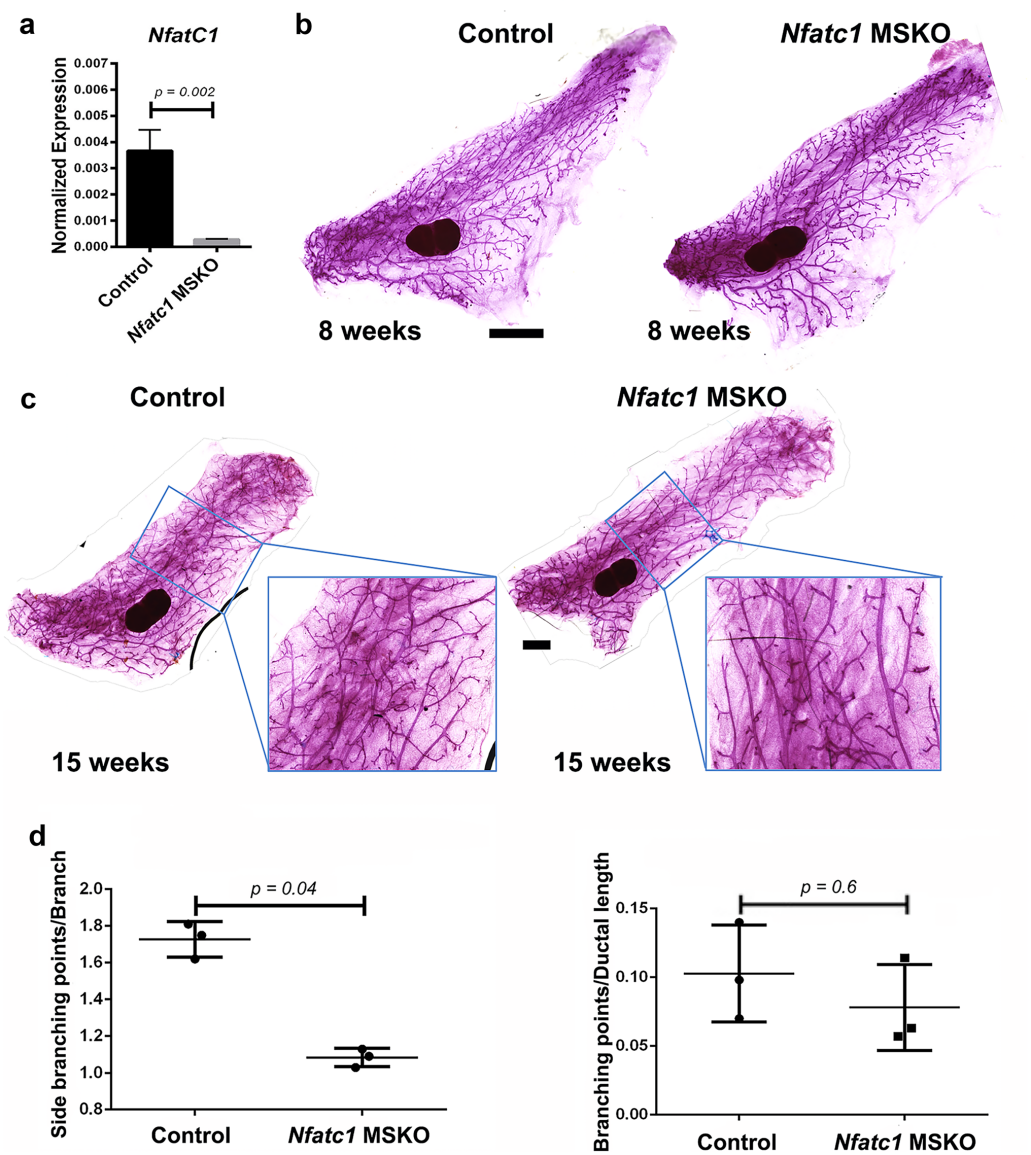
Mammary glands of *Nfatc1*-deficient mice exhibit mild defects at maturation. **a**, RT-qPCR analysis of sorted mammary basal cells from 15-week-old virgin *Nfatc1* MSKO and control (*K14-Cre;Nfatc1*^*f/*+^) littermates. N = 3. **b-c**, Representative whole-mount carmine staining images of mammary glands from 8-week-old (**b**) and 15-week-old (**c**) virgin *Nfatc1* MSKO and control (*K14-Cre;Nfatc1*^*f/*+^) littermates. Boxed areas in **c** are similarly enlarged for control and mutant. **d**, Quantification of the numbers of alveolar buds/side branches (left) and major branches (right) from N = 3 pairs of 15-week-old virgin *Nfatc1* MSKO and control (*K14-Cre;Nfatc1*^*f/*+^ or *Nfatc1*^*f/f*^) littermates. Each dot or square represents a single mouse. Scale bar: 3 mm in **b**, **c**.

To examine the physiological function of *Nfatc1* in mammary epithelial cells, we used *K14-Cre* as a driver to generate mammary epithelial-specific knockout (MSKO) of *Nfatc1 (K14-Cre;Nfatc1*^*f/f*^*)*. RT-qPCR analysis of control and MSKO basal cells confirmed the reduction of *Nfatc1* mRNA expression in the latter (Fig. 2a). At 8 weeks of age, mammary glands from *Nfatc1* MSKO mice were similar in morphology to control littermate counterparts (Fig. 2b), suggesting that pubertal mammary development is largely intact. However, by 15 weeks of age, the number of alveolar buds and side branches was significantly reduced in *Nfatc1* MSKO mice, whereas the number of major branches was not significantly affected (Fig. 2c, d). This finding suggests a minor role for *Nfatc1* in promoting robust mammary epithelial maturation.

**Fig. 3:**
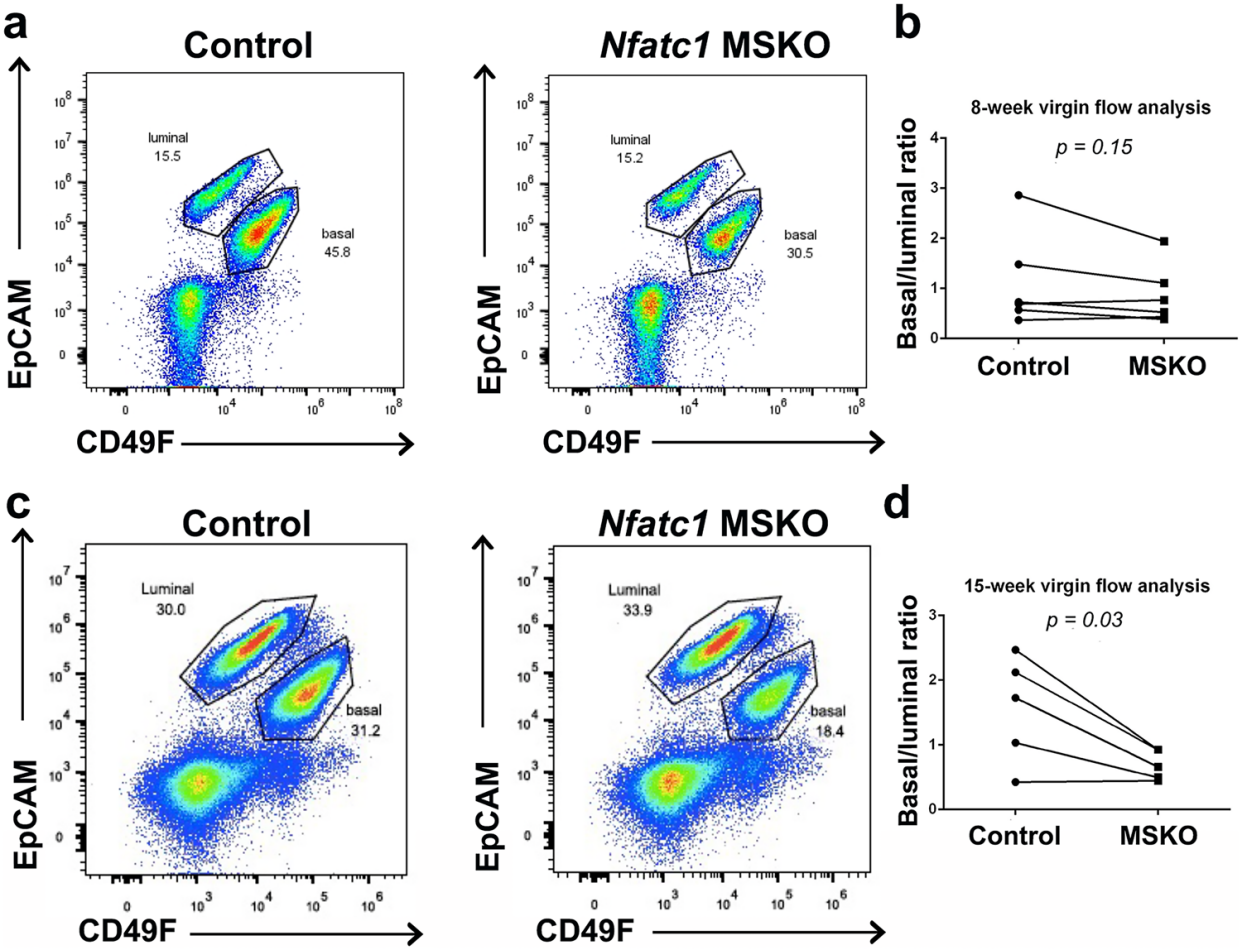
*Nfatc1* deficiency affects mammary basal-luminal balance. Shown are representative flow cytometry profiles (**a**, **c**) and quantification of the ratio between basal and luminal cells **(b, d)** in mammary glands from 8-week-old (**a**, **b**) and 15-week-old (**c**, **d**) *Nfatc1* MSKO and control (*Nfatc1*^*f/f*^ or *Nfatc1*^*f/*+^) littermates. N >  = 3 pairs. Each dot or square represents a single mouse. Lines between a dot and a square indicate littermates.

The specific expression of *Nfatc1* in basal cells, plus previous report that basal/luminal cell ratio affects side branching [23], led us to wonder whether *Nfatc1* deletion affects the balance between basal and luminal cells. While the ratio between flow-detected basal and luminal cell populations was similar in mammary glands from 8-week-old virgin *Nfatc1* MSKO and control littermates (Fig. 3a, b), a significant decrease in basal/luminal ratio was observed in mammary glands from 15-week-old *Nfatc1* MSKO virgin females compared to control littermates (Fig. 3c, d). Thus, loss of *Nfatc1* in the mammary epithelium skews the balance between basal and luminal cells towards the latter, but this alteration is only obvious at mammary epithelial maturation when the morphological defect also manifests.

### *Nfatc1* Confers Maximal in Vitro Clonogenicity and in Vivo Regenerative Potential to Mammary Basal Cells

**Fig. 4:**
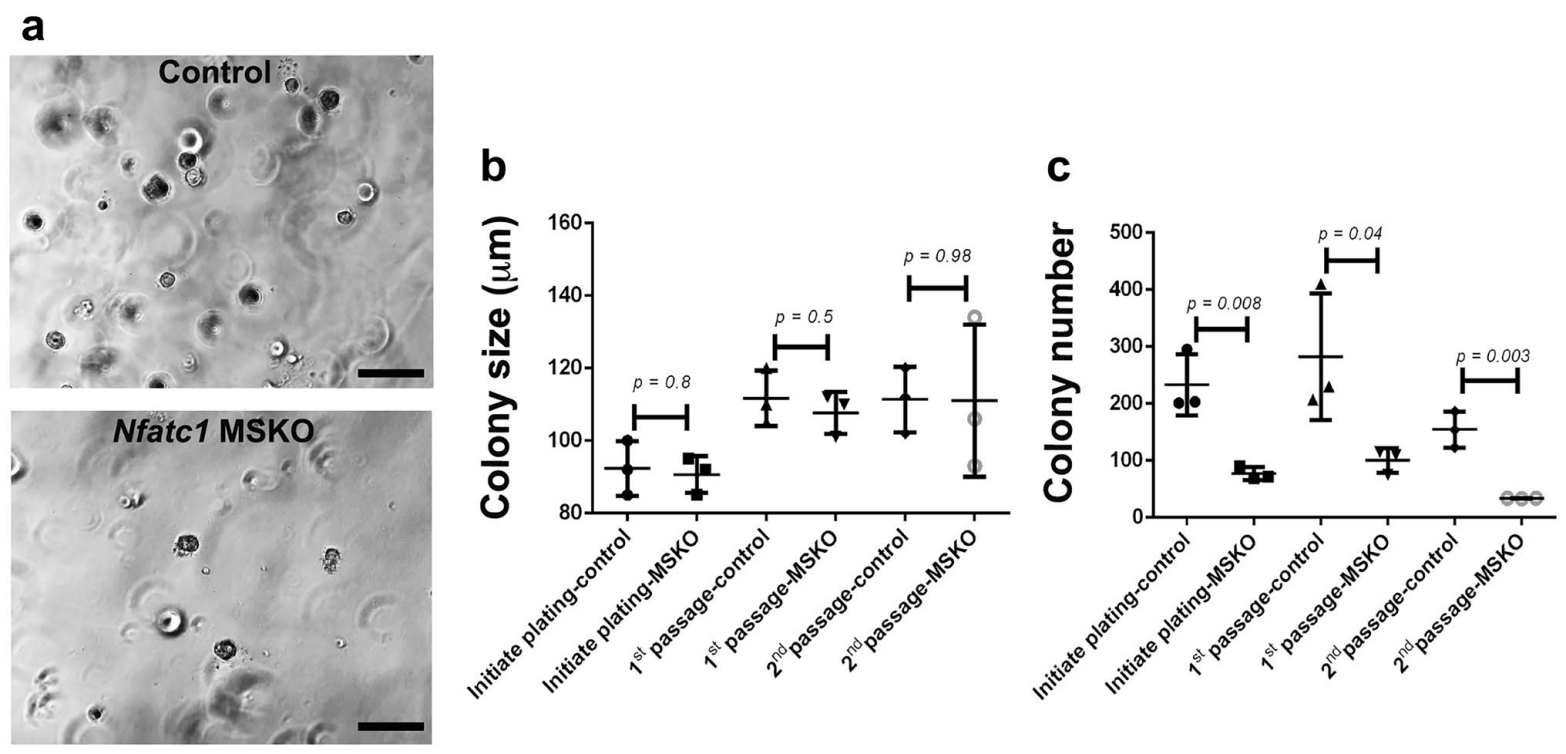
*Nfatc1*-deficient basal cells show significantly reduced colony formation potential in vitro. **a**, Representative images of colonies derived from FACS-sorted basal cells from 8-week-old *Nfatc1* MSKO and control (*K14-Cre;Nfatc1*^*f/*+^, *Nfatc1*^*f/f*^, or *Nfatc1*^*f/*+^) littermates. Scale bar: 500 μm. **b-c**, Quantification of colony size (**b**) and number (**c**) for basal cells derived from N = 3 pairs of *Nfatc1* MSKO and control (*K14-Cre;Nfatc1*^*f/*+^, *Nfatc1*^*f/f*^, or *Nfatc1*^*f/*+^) littermates. Colonies from initial plating were harvested and equal numbers (5,000) of *Nfatc1* MSKO and control cells were subsequently passaged once (1^st^ passage) or twice (2^nd^ passage). Each dot, square, or triangle represents cells derived from a single mouse.

To ask whether *Nfatc1* loss specifically impacts mammary basal cells, we performed ex vivo 3D colony formation assays by culturing FACS-sorted basal cells from *Nfatc1* MSKO and control littermates in Matrigel [24–27]. Colonies formed by the *Nfatc1* MSKO basal cells were similar in size to those formed by control counterparts both in initial plating and when the cells were serially passaged (Fig. 4a, b). However, at all passages examined, the number of colonies formed in the *Nfatc1* MSKO culture was significantly lower than that in the control culture (Fig. 4c). These results suggest that *Nfatc1* likely regulates the self-renewal capacity of mammary basal stem/progenitor cells, rather than the rate of basal cell proliferation per se.

**Fig. 5:**
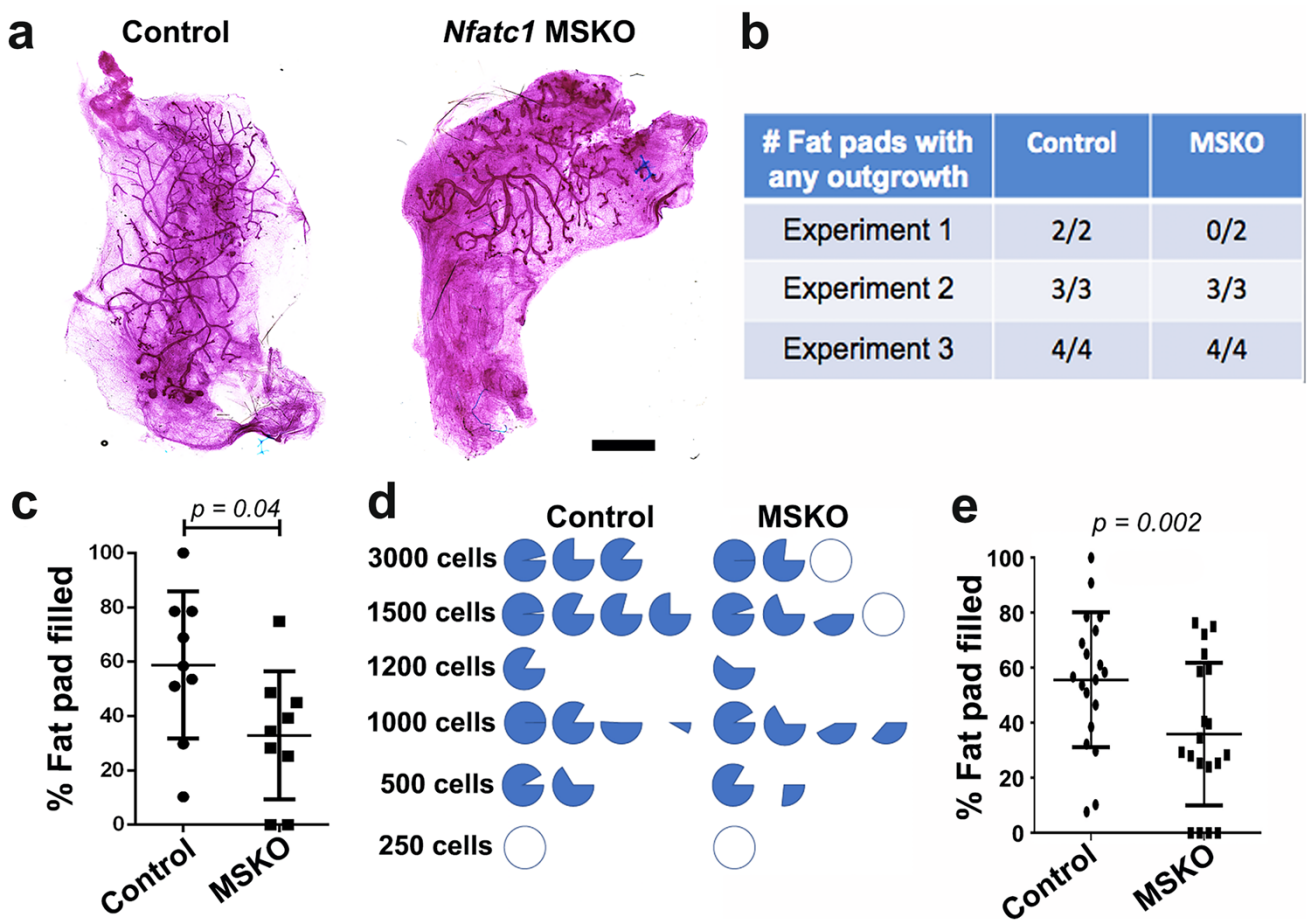
*Nfatc1*-deficient basal cells show reduced regenerative capacity in vivo. **a-b**, Representative transplants (**a**) derived from basal cells isolated from 8-week-old *Nfatc1* MSKO and control (*K14-Cre;Nfatc1*^*f/*+^, *Nfatc1*^*f/f*^, or *Nfatc1*^*f/*+^) littermates, and summary of take rate (producing a tree that is > 5% of the fat pad) from 3 different experiments (**b**). Each experiment included 1 pair of *Nfatc1* MSKO and control (*K14-Cre;Nfatc1*^*f/*+^, *Nfatc1*^*f/f*^, or *Nfatc1*^*f/*+^) littermate as donor mice, and 3,000 basal cells from each were transplanted onto the contralateral sides of the same host mice. Scale bar: 3 mm. **bc**, Summary of percent of fat pad filled from (**b**). **d-e**, Summary of results from limiting dilution transplantations using FACS-sorted mammary basal cells from additional *Nfatc1* MSKO and control (*K14-Cre;Nfatc1*^*f/*+^, *Nfatc1*^*f/f*^, or *Nfatc1*^*f/*+^) littermates. Each pie diagram (**d**) represents an outgrowth, and data for percent of fat pad filled are summarized in (**e**). Each dot or square in (**c**, **d**) represents a single recipient mouse.

To complement the in vitro findings above, we conducted transplantation experiments by injecting FACS-sorted basal cells from *Nfatc1* MSKO and control littermates into de-epithelialized fat pads of congenic 3-week-old host mice. In three independent experiments where 3,000 *Nfatc1* MSKO or control basal cells were injected, the take rate appeared to be minimally impacted (9 out of 9 transplants in control vs. 7 out of 9 in MSKO), but a significant reduction in the percent of fat pad filling was observed in outgrowths derived from the latter (Fig. 5a–c). In separate transplantation experiments using serially decreasing numbers of basal cells from additional pairs of donor mice, the overall take rate was only slightly lower but the average percent of fat pad filling by *Nfatc1* MSKO basal cells was again significantly reduced compared to control basal cells (Fig. 5d, e). These results show that *Nfatc1* plays a detectable, albeit non-essential, role in promoting the in vivo regenerative potential of mammary basal cells.

## Discussion

Our work has uncovered a role of *Nfatc1* in regulating mammary epithelial morphogenesis. The mammary phenotypes caused by epithelial-specific knockout of *Nfatc1* are mild, with morphological and basal/luminal ratio defects becoming apparent only in adulthood. As such, *Nfatc1* is largely dispensable in the mammary epithelium for ductal elongation and initial branching morphogenesis during pubertal development.

Our findings provide evidence for *Nfatc1*’s function in promoting mammary basal stem/progenitor cell self-renewal. Of note, even though no morphological defect was detectable in *Nfatc1* MSKO mice at 8 weeks of age, basal cells isolated at this developmental stage showed reduced colony forming and regenerative capabilities. This implicates the inherent deficiency of *Nfatc1*-deficient basal cells that can be unmasked when cells are taken out of their endogenous tissue environment, consistent with the prevailing theme of differential basal stem cell potential under physiological vs. heterogeneous environments [5, 7–9, 28, 29]. *Nfatc1*-deficient basal cells may lose their self-renewal capability because of compromised cell adhesion, basal gene expression, and/or proliferation potential. Future work is needed to address these possibilities.

It is somewhat surprising that *Nfatc1* expression in such a small number of basal cells (~ 2% at 8 weeks) in vivo translates into a detectable minor branching phenotype. It is possible that *Nfatc1*-expressing basal cells might represent a pool of stem/progenitor cells, where *Nfatc1* functions cell-autonomously, in the basal compartment that contribute to side branching and alveolar bud formation. Lineage tracing analysis using *Nfatc1-CreER* mice, which lies outside the scope of the current work, will be able to track the fate of the *Nfatc1*-expressing basal cells during mammary epithelial development and regeneration. Alternatively, *Nfatc1* might regulate the expression of genes encoding secreted factors, which in turn impact a large number of cells including the non-*Nfatc1*-expressing basal cells.

Our findings add *Nfatc1* to the growing list of regulatory factors that govern the activities of mammary basal stem/progenitor cells. High levels of NFATc1 expression in breast cancer correlate with poor prognosis [14]. The functional involvement of *Nfatc1* in normal mammary basal stem/progenitor cells raises the possibility that NFATc1 may also regulate the activity of malignant stem/progenitor cells in breast cancer, a notion worth testing in the future.

## Materials and Methods

### Mice

*Nfatc1*^f/f^ mice [30] were purchased from the Jackson Laboratory (Stock # 022786) and bred with *K14-Cre* mice [31, 32] to generate mammary epithelial-specific knockout (MSKO) of *Nfatc1*. *Nfatc1*-Cre mice were as previously reported [33], and were bred with *ROSA*^mTmG^ mice the from the Jackson Laboratory (Stock # 007576). Primers used for genotyping are listed in Table S1. Control and mutant littermates were housed in the same cage before analysis to synchronize the estrous cycles [34]. All mouse experiments have been approved by and conform to the regulatory guidelines of the International Animal Care and Use Committee of the University of California, Irvine.

### Isolation of Mammary Epithelial Cells

Single-cell preparation of mammary epithelial cells was performed as previously described [22, 32]. Briefly, mammary glands were isolated from 8-week-old virgin or other specified-stage female mice and placed in the digestion mix [DMEM/F12 (1:1) with 5% FBS, 300 U/mL collagenase (Sigma, C9891) and 100 U/mL hyaluronidase (Sigma, H3506)] for 1.5 h at 37 °C. Cells were pelleted and resuspended in red blood cell lysis buffer (Sigma, R7757). Single cell suspension was obtained by further treatment with 0.25% trypsin–EDTA (Gibco, 25200), 10 mg/mL DNase (Sigma, DN25), and 5 mg/mL dispase (Stem Cell Technologies, 07913), followed by filtration using a 40 μm-pore mesh filter (SWiSH, TC70-MT-1).

### Cell Labeling and Flow Cytometry

Single cell suspension from above was stained using the following antibodies and reagents: anti-CD49f-FITC (1:250, Bio Legend, 102205), anti-EpCAM-PE-Cy7 (1:250, Bio Legend, 118215), anti-lineage-APC [1:250; including APC-CD45 (BD Biosciences, 559864), APC-CD31 (BD Biosciences, 551262), APC-TER119 (BD Biosciences, 557909)], and SytoxBlue (Invitrogen, S3457). Flow cytometry analysis and sorting were performed on a FACSAria (Becton Dickenson UK).

### Gene Expression Analysis

Total RNA was extracted from the FACS-sorted cells using RNeasy Mini Kit with on-column DNase treatment according to manufacturer’s protocol (QIAGEN). cDNA was synthesized using High-Capacity cDNA Reverse Transcription Kit (Thermo Fisher Scientific) according to manufacturer’s instructions. Real-time PCR was performed using a SYBR Green Supermix (BioRad) on a CFX96 RT-qPCR system, and data were analyzed using the 2 − ΔΔCT method. Primers used for qPCR are listed in Table S1. Analysis of *Nfatc1* expression during pregnancy and lactation was based on a published microarray dataset (https://www.ncbi.nlm.nih.gov/geoprofiles/42110332) on mammary gland samples from FVB mice [19, 20].

### Immunohistochemistry and Mammary Gland Whole-mount Analysis

Immunohistochemistry was performed as previously described [32]. For whole-mount analysis, the #4 pair of mammary glands were dissected and fixed in Carnoy’s fixative (10% acetic acid, 30% CHCl3, 60% ethanol) for 2–4 h. Fixed tissues were treated with a gradient of ethanol (100%, 70%, 30%) and then washed with sterile water for 10 min. Tissues were then incubated with carmine-alum staining solution as previously described [35]. Images were captured using a Keyence microscope.

### Cleared Fat Pad Transplantation

Fat pad clearing and transplantation was as previously described [32]. Briefly, single cell suspensions of sorted basal cells from *Nfatc1* MSKO and control littermates were prepared as described above and diluted in a 1:1 solution of 5% FBS media/Matrigel at a concentration of 2,000 cells/10 μL. Ten μL of the cell/Matrigel solution was injected into cleared fat pads of #4 mammary glands of 3-week-old C57BL/6 females, with each host mouse receiving contralateral injections of MSKO and control samples. Outgrowths were analyzed 8–9-weeks later using whole-mount carmine alum staining.

### Colony Formation

Sorted basal cells were resuspended in a chilled 1:1 solution of EpiCult-B medium (Stem Cell Technologies)/growth factor-reduced Matrigel (BD Biosciences), and 5,000 cells were plated into one well from 8-well chamber slides (Thermo Fisher Scientific). After Matrigel hardens, 400 μl of EpiCult-B medium containing 10 ng/mL EGF (Millipore, 01–107), 10 ng/mL bFGF (PeproTech, 100-18B), and 4 μg/mL heparin (Stem Cell Technologies, 07980) was added into each well. Culture medium was changed every three days for two weeks, followed by counting colony number and measuring colony size. To passage the colonies, medium was first removed and Matrigel dissolved using 200 μL of dispase for at least 20 min. Single cells were obtained by incubation in 0.25% Trypsin–EDTA followed by filtration using a 40 μm filter, and 5,000 cells were then re-plated as described above.

### Statistics

Independent experiments were performed on at least three biological replicates. The sample sizes are indicated in the relevant figure legends. For analysis of differences between groups, Student’s paired *t*-test was performed with Prism. *p* values of 0.05 or less were considered statistically significant. Error bars in figures represent mean ± standard error of the mean (SEM).

## Supplementary Information

Below is the link to the electronic supplementary material.

Supplementary file1 (DOCX 194 KB)

## References

[1] Fu NY, Nolan E, Lindeman GJ, Visvader JE. Stem cells and the differentiation hierarchy in mammary gland development. Physiol Rev. 2020. 10.1152/physrev.00040.2018 31539305

[2] Macias H, Hinck L. Mammary gland development. Wiley Interdiscip. Rev Dev Biol. 2012. 10.1002/wdev.35 PMC3404495 22844349

[3] Sternlicht MD, Kouros-Mehr H, Lu P, Werb Z. Hormonal and local control of mammary branching morphogenesis. Differentiation. 2006. 10.1111/j.1432-0436.2006.00105.x PMC2580831 16916375

[4] Watson CJ, Khaled WT. Mammary development in the embryo and adult: A journey of morphogenesis and commitment. Development. 2008. 10.1242/dev.005439 18296651

[5] Rios AC, Fu NY, Lindeman GJ, Visvader JE. In situ identification of bipotent stem cells in the mammary gland. Nature. 2014. 10.1038/nature12948 24463516

[6] Visvader JE, Stingl J. Mammary stem cells and the differentiation hierarchy: Current status and perspectives. Genes Dev. 2014. 10.1101/gad.242511.114 PMC4052761 24888586

[7] Van Keymeulen A, Rocha AS, Ousset M, Beck B, Bouvencourt G, Rock J, et al. Distinct stem cells contribute to mammary gland development and maintenance. Nature. 2011. 10.1038/nature10573 21983963

[8] Shackleton M, Vaillant F, Simpson KJ, Stingl J, Smyth GK, Asselin-Labat ML, et al. Generation of a functional mammary gland from a single stem cell. Nature. 2006. 10.1038/nature04372 16397499

[9] Stingl J, Eirew P, Ricketson I, Shackleton M, Vaillant F, Choi D, et al. Purification and unique properties of mammary epithelial stem cells. Nature. 2006. 10.1038/nature04496 16395311

[10] Crabtree GR, Olson EN. NFAT signaling: Choreographing the social lives of cells. Cell. 2002. 10.1016/s0092-8674(02)00699-2 11983154

[11] Li X, Zhu L, Yang A, Lin J, Tang F, Jin S, et al. Calcineurin-NFAT signaling critically regulates early lineage specification in mouse embryonic stem cells and embryos. Cell Stem Cell. 2011. 10.1016/j.stem.2010.11.027 21211781

[12] Horsley V, Aliprantis AO, Polak L, Glimcher LH, Fuchs E. NFATc1 Balances Quiescence and Proliferation of Skin Stem Cells. Cell. 2008. 10.1016/j.cell.2007.11.047 PMC2546702 18243104

[13] Lee JH, Bhang DH, Beede A, Huang TL, Stripp BR, Bloch KD, et al. Lung stem cell differentiation in mice directed by endothelial cells via a BMP4-NFATc1-thrombospondin-1 axis. Cell. 2014. 10.1016/j.cell.2013.12.039 PMC3951122 24485453

[14] Kang X, Wang S, Song Y. NFATc1 protein expression and its relationship with clinical characteristics in breast cancer. J Clin Oncol. 2012.

[15] Oikawa T, Nakamura A, Onishi N, Yamada T, Matsuo K, Saya H. Acquired expression of NFATc1 downregulates e-cadherin and promotes cancer cell invasion. Cancer Res. 2013. 10.1158/0008-5472.CAN-13-0274 23811942

[16] Tran Quang C, Leboucher S, Passaro D, Fuhrmann L, Nourieh M, Vincent-Salomon A, et al. The calcineurin/NFAT pathway is activated in diagnostic breast cancer cases and is essential to survival and metastasis of mammary cancer cells. Cell Death Dis. 2015. 10.1038/cddis.2015.14 PMC4669815 25719243

[17] Kessenbrock K, Dijkgraaf GJP, Lawson DA, Littlepage LE, Shahi P, Pieper U, et al. A Role for matrix metalloproteinases in regulating mammary stem cell function via the Wnt signaling pathway. Cell Stem Cell. 2013. 10.1016/j.stem.2013.06.005 PMC3769456 23871604

[18] Yiu GK, Toker A. NFAT induces breast cancer cell invasion by promoting the induction of cyclooxygenase-2. J Biol Chem. 2006. 10.1074/jbc.M600184200 16505480

[19] Rudolph MC, McManaman JL, Hunter L, Phang T, Neville MC. Functional development of the mammary gland: Use of expression rofiling and trajectory clustering to reveal changes in gene expression during pregnancy, lactation, and involution. J Mammary Gland Biol Neoplasia. 2003. 10.1023/b:jomg.0000010030.73983.57 14973374

[20] Anderson SM, Rudolph MC, McManaman JL, Neville MC. Key stages in mammary gland development. Secretory activation in the mammary gland: It’s not just about milk protein synthesis!. Breast Cancer Res. 2007. 10.1186/bcr1653 PMC1851396 17338830

[21] Soady KJ, Kendrick H, Gao Q, Tutt A, Zvelebil M, Ordonez LD, et al. Mouse mammary stem cells express prognostic markers for triple-negative breast cancer. Breast Cancer Res. 2015. 10.1186/s13058-015-0539-6 PMC4381533 25849541

[22] Gu B, Watanabe K, Sun P, Fallahi M, Dai X. Chromatin effector Pygo2 mediates wnt-notch crosstalk to suppress luminal/alveolar potential of mammary stem and basal cells. Cell Stem Cell. 2013;13. 10.1016/j.stem.2013.04.012 PMC3703489 23684539

[23] Macias H, Moran A, Samara Y, Moreno M, Compton JE, Harburg G, et al. SLIT/ROBO1 signaling suppresses mammary branching morphogenesis by limiting basal cell number. Dev Cell. 2011. 10.1016/j.devcel.2011.05.012 PMC3129866 21664580

[24] Jardé T, Lloyd-Lewis B, Thomas M, Kendrick H, Melchor L, Bougaret L, et al. Wnt and Neuregulin1/ErbB signalling extends 3D culture of hormone responsive mammary organoids. Nat Commun. 2016. 10.1038/ncomms13207 PMC5095178 27782124

[25] Gu B, Watanabe K, Sun P, Fallahi M, Dai X (2013). Chromatin effector Pygo2 mediates wnt-notch crosstalk to suppress luminal/alveolar potential of mammary stem and basal cells. Cell Stem Cell.

[26] Watanabe K, Villarreal-Ponce A, Sun P, Salmans ML, Fallahi M, Andersen B, et al. Mammary morphogenesis and regeneration require the inhibition of EMT at terminal end buds by ovol2 transcriptional repressor. Dev Cell. 2014;29. 10.1016/j.devcel.2014.03.006 PMC4062651 24735879

[27] Kleinman HK, Martin GR. Matrigel: Basement membrane matrix with biological activity. Semin Cancer Biol. 2005. 10.1016/j.semcancer.2005.05.004 15975825

[28] Prater MD, Petit V, Alasdair Russell I, Giraddi RR, Shehata M, Menon S, et al. Mammary stem cells have myoepithelial cell properties. Nat Cell Biol. 2014. 10.1038/ncb3025 PMC4183554 25173976

[29] Van Amerongen R, Bowman AN, Nusse R. Developmental stage and time dictate the fate of Wnt/β-catenin- responsive stem cells in the mammary gland. Cell Stem Cell. 2012. 10.1016/j.stem.2012.05.023 PMC13155203 22863533

[30] Aliprantis AO, Ueki Y, Sulyanto R, Park A, Sigrist KS, Sharma SM, et al. NFATc1 in mice represses osteoprotegerin during osteoclastogenesis and dissociates systemic osteopenia from inflammation in cherubism. J Clin Invest. 2008. 10.1172/JCI35711 PMC2564610 18846253

[31] Gu B, Sun P, Yuan Y, Moraes RC, Li A, Teng A (2009). Pygo2 expands mammary progenitor cells by facilitating histone H3 K4 methylation. J Cell Biol.

[32] Watanabe K, Villarreal-Ponce A, Sun P, Salmans ML, Fallahi M, Andersen B, et al. Mammary morphogenesis and regeneration require the inhibition of EMT at terminal end buds by ovol2 transcriptional repressor. Dev Cell [Internet]. Elsevier Inc.; 2014;29:59–74. Available from: 10.1016/j.devcel.2014.03.006. 10.1016/j.devcel.2014.03.006 PMC4062651 24735879

[33] Wu B, Zhang Z, Lui W, Chen X, Wang Y, Chamberlain AA, et al. Endocardial cells form the coronary arteries by angiogenesis through myocardial-endocardial VEGF signaling. Cell. 2012. 10.1016/j.cell.2012.10.023 PMC3508471 23178125

[34] Handelmann G, Ravizza R, Ray WJ. Social dominance determines estrous entrainment among female hamsters. Horm Behav. 1980. 10.1016/0018-506x(80)90002-1 7191403

[35] Sympson CJ, Talhouk RS, Alexander CM, Chin JR, Clift SM, Bissell MJ, et al. Targeted expression of stromelysin-1 in mammary gland provides evidence for a role of proteinases in branching morphogenesis and the requirement for an intact basement membrane for tissue-specific gene expression. J Cell Biol. 1994. 10.1083/jcb.125.3.681 PMC2119999 8175886

