# Supplementary material for: Nfatc1’s Role in Mammary Epithelial Morphogenesis and Basal Stem/progenitor Cell Self-renewal
Source: J Mammary Gland Biol Neoplasia. 2021 Dec 21;26(4):357–65. doi: 10.1007/s10911-021-09502-6 (PMC8858291; doi:10.1007/s10911-021-09502-6)
Supplement: Supplementary file 1 — Supplementary file1 (DOCX 194 KB) [file 10911_2021_9502_MOESM1_ESM.docx]

***Nfatc1*’s role in mammary epithelial morphogenesis and basal stem/progenitor cell self-renewal**

Melissa McNeil, Yingying Han, Peng Sun, Kazuhide Watanabe, Jun Jiang, Zhengquan Yu, Bin Zhou, and Xing Dai

**SUPPLEMENTAL TABLE**

| **Supplemental Table 1. Information of primers used in the study.** | | |
| --- | --- | --- |
| *Primers for RT-qPCR* |  |  |
| Primer | Allele detected | Sequence |
| Forward control | *Nfatc1 +,* f, and - alleles | AAGGAATTACTGGGAAGCCTGGCA |
| Forward mutant |  | AGGGACTATCATTTGGCAGGGACA |
| Reverse |  | ACAGGAAACAGCTCTGTTCCACAC |
| Forward | *Nfatc1* f and + alleles | GGACAGTCTAAGGCCTGCTG |
| Reverse |  | ACCCCACATCCCAGAGTGA |
| Forward | *Nfatc1-Cre* allele | GAAGCAACTCATCGATTGATTTACG |
| Reverse |  | AACCCTGGACGCCTGGGACAC |
| *Primers for RT-qPCR* |  |  |
| Forward | *GAPDH* cDNA | CCTGCCAAGTATGATGAC |
| Reverse |  | GGAGTTGCTGTTGAAGTC |
| Forward | *Nfatc1* cDNA | CCCGTCACATTCTGGTCCAT |
| Reverse |  | CAAGTAACCGTGTAGCTGCACAA |

**SUPPLEMENTAL FIGURE**

**
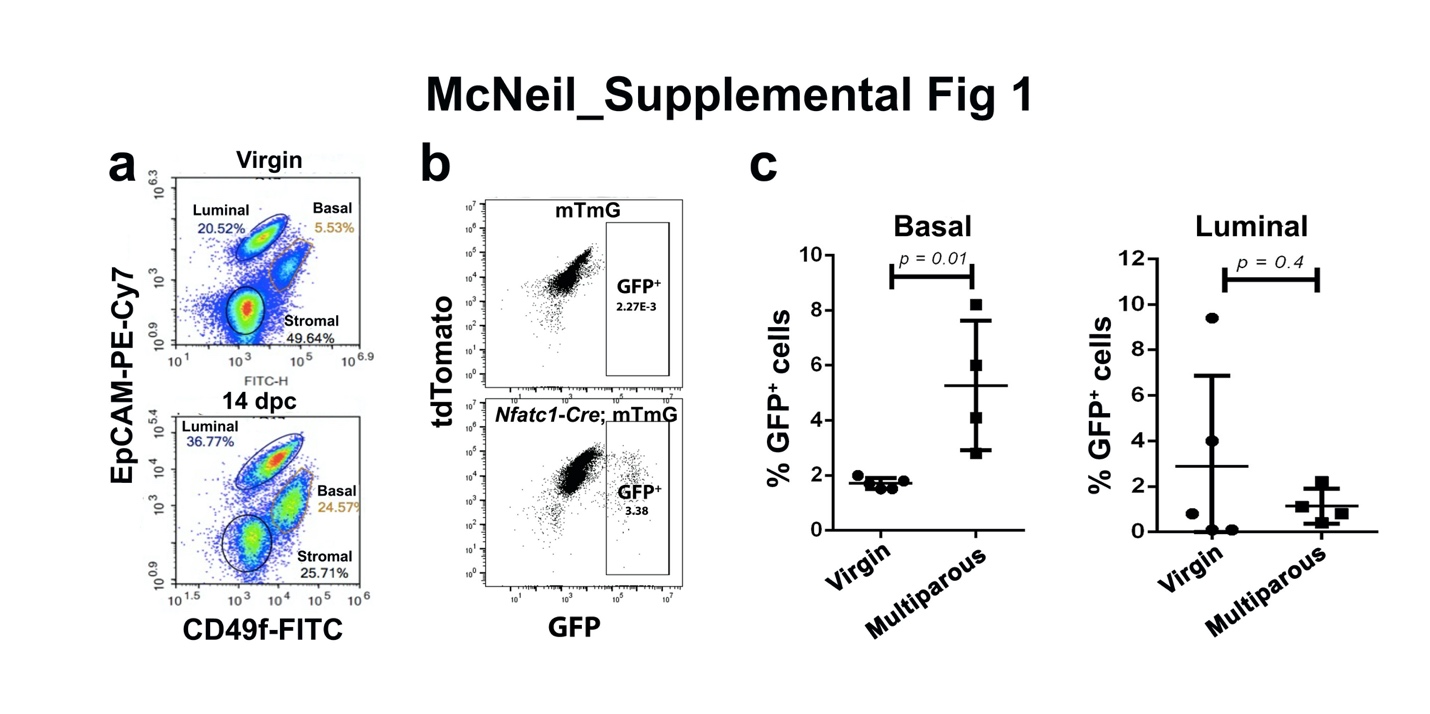
**

**Figure S1. Supplemental data for Figure 1.** a, Representative flow cytometry profiles for basal and luminal cells from 8-week-old virgin and 15-week-old mid-pregnant (P14) WT female mice. b, Flow cytometry profiles to show the presence of GFP^+^ mammary basal cells in multiparous (3 pregnancies) *Nfatc1-Cre;ROSA^mTmG^* female mice, but not *ROSA^mTmG^* control littermates. c, Quantification of GFP^+^ cells in the basal and luminal cell populations of mammary glands of 8-week-old virgin *Nfatc1-Cre;ROSA^mTmG^* females (N=5) or 6-9-month-old multiparous *Nfatc1-Cre;ROSA^mTmG^* mice (N=4).
